# Integrating marker-assisted backcross breeding and genomic analysis to obtain thermotolerant tomato genotypes for the fresh market

**DOI:** 10.1007/s11032-026-01730-0

**Published:** 2026-10-29

**Authors:** Salvatore Graci, Matteo Addonizio, Dario Giglio, Silvana Francesca, Amalia Barone

**Affiliations:** 1 Department of Agricultural Science, University of Naples Federico II, Via Università 100, Portici, 80055 Italy; 2 Plant Breeding, Wageningen University & Research (WUR), Wageningen, 6708 PB the Netherlands

**Keywords:** Tomato, Heat stress, Marker assisted selection, Next generation sequencing, Linkage drag

## Abstract

Current climate change significantly impacts on agriculture, negatively affecting plant growth and development of many agricultural crops including tomato. To adapt to current increase in temperatures, it is essential to develop novel heat tolerant cultivars. The present study describes a backcross breeding program developed to produce new genotypes suitable for the fresh tomato market that exhibit the favorable characteristics of the previously selected thermotolerant genotype E42. This was achieved by converting the determinate plant habitus of E42 to indeterminate. The SP_CAPS marker designed to target a SNP in the *self-pruning* gene, which is responsible for the plant habitus, enabled us to accelerate the selection of indeterminate plants, thus obtaining nine BC_4_S_1_ lines. The evaluation of ten phenotypic traits in two different growth conditions (open field and under plastic tunnel) evidenced the good performances of the BC_4_S_1_ lines and the phenotypic recovery of the recurrent parent in all of them. Interestingly, one genotype exceeded the mean yield values of the group when grown in open field. In addition, the analysis of the ddRAD sequencing data revealed a high level of genomic recovery, with only a few variants retained from the donor parents. Clear linkage drag was assessed in almost all the BC_4_S_1_ lines around the *self-pruning* gene that was targeted using Marker Assisted Selection. Altogether, this work provided improved thermotolerant genotypes with high fruit quality that may find place in the tomato fresh market.

**Supplementary Information:**

The online version contains supplementary material available at https://doi.org/10.1007/s11032-026-01730-0.

## Introduction

Tomato (*Solanum lycopersicum L.*) is one of the most important vegetables produced all over the world and is a highly relevant economically species, being the second horticultural crop in terms of cultivated area. Two main commercial groups can be identified: (I) the fresh market is intended for direct consumption (Bhandari et al. 2023); (II) the processing tomato is destined for processed foods (Guan et al. 2018). The tomato fresh market includes a wide diversity of fruits that differ in size (from 10 g to more than 150 g), shapes, color, and taste (Tanksley 2004). Most of this plant material are F_1_ hybrids or landraces found in the Mediterranean area, which represents the secondary center of diversification of the species (Cebolla-Cornejo et al. 2013; Caramante et al. 2021; Graci et al. 2022). Modern varieties have been improved through the development of breeding programs for tolerance to abiotic stresses, resistance to diseases, postharvest shelf-life and some sensory and nutritional traits (Casals et al. 2018; Schouten et al. 2019). Current climate changes caused by rising temperatures are predicted to significantly affect plant growth and development, comporting yield losses and altered fruit quality (Bita and Gerats 2013). Heat stress (HS) has negative effects on plant morphology, development, physiology, biochemistry, and molecular pathways at vegetative and reproductive stages (Alsamir et al. 2021; Hoshikawa et al. 2021; Riccini et al. 2025). High temperatures in tomato fields mainly occur during the reproductive stages, particularly in the Mediterranean area, causing flower abscission, impaired growth of stamens and pistils, poor pollen germination and altered pollen tube development with consequent low levels of fruit set and losses in the final yield (Ayenan et al. 2019; Raja et al. 2019; Agho et al. 2024; Graci and Barone 2024). In this context, identifying heat-tolerant tomato genotypes is a key challenge for breeders to mitigate the impact of abiotic stresses and reduce yield losses. During the last years, we selected a thermotolerant genotype named E42, which exhibited high productive and fruit quality traits under high temperature conditions (Ruggieri et al. 2019; Olivieri et al. 2020, 2021; Francesca et al. 2024; Graci et al. 2024, 2025). It is a cherry tomato type that produces clusters of more than 10 fruits, compact and suitable for fresh consumption. However, its determinate plant habitus makes it unsuitable for the fresh market, since determinate plants are usually harvested mechanically at the same time and therefore are mostly intended for the processed market. To allocate E42 for the fresh market, and therefore with scalar harvest, this genotype must be transformed from determinate to indeterminate, while retaining all its other desirable traits. The backcross breeding method is one of the strategies developed to improve one trait of an elite genotype. This method has been extensively used in plant breeding to incorporate into an adapted or elite variety (the recurrent parent) one or a few genes from a genotype with a unique trait (the donor parent) (Mutlu et al. 2005; Vasistha et al. 2016; Lee et al. 2018; Janaki Ramayya et al. 2021). However, this method is time-consuming and relatively expensive (Kabelka et al. 2004; Osei et al. 2018). To speed up the backcross scheme, the use of marker-assisted selection (MAS) has been successfully exploited in many breeding programs (Dreher et al. 2003; Collard and Mackill 2008; Foolad and Panthee 2012; Lenaerts et al. 2019). This molecular tool enables selection to be applied at an early stage of plant development, reducing time and costs (Hwang et al. 2012; Hasan et al. 2015; Kim et al. 2019). However, since the target gene is linked to its neighboring loci, selection at the target locus maintains the donor genome around this locus in a phenomenon known as linkage drag (Lyzenga et al. 2021; Tourrette et al. 2021).

The present work aimed at developing a backcross breeding program to modify the plant habitus of the E42 genotype from determinate to indeterminate, thus enabling it to be considered in the future as a variety for the fresh tomato market. Therefore, the proposed backcross program aimed to transfer the dominant allele (*Sp*) of the *self-pruning* gene (SP) to the E42 genotype. This allele indeed confers an indeterminate habitus to the plant (Pnueli et al. 1998). To this end, a specific molecular marker has been designed in the present work to carry out a Molecular Assisted Backcross (MABC) scheme. Additionally, phenotypic evaluations were conducted to assess the performance of the final BC_4_S_1_ lines obtained under two different growth conditions. The recovery of the recurrent genome in these lines was also evaluated using the high-throughput genotyping ddRAD sequencing platform.

## Materials and methods

### Plant material

Five tomato indeterminate genotypes (PL1, PL2, PL3, PL4, PL5) from a tomato germplasm collection available at the University of Naples Federico II, Department of Agricultural Sciences (details hosted in the LabArchive repository, https://doi.org/10.6070/H4TT4NXN) were chosen as donors for carrying out the backcross breeding program with the recurrent E42 genotype (Table S1). They were previously selected for their good production and fruit quality performances when cultivated under high temperature conditions (Ruggieri et al. 2019; Olivieri et al. 2020, 2021), and exhibited small fruit size (10–30 g) and different shape (from circular to cylindric) (Fig. S1).

### Molecular marker design

Total genomic DNA was extracted from leaves harvested from seedlings, using a slightly modified protocol of the CTAB method (Doyle and Doyle 1987) reported by Graci et al. (2022). The allelic condition of the *self-pruning* gene (*Solyc06g074350*) was investigated by designing the Cleaved Amplified Polymorphic Sequence (CAPS) SP_CAPS marker on a Single Nucleotide Polymorphism (SNP) mutation identified by Pnueli et al. (1998). PCR was carried out in 25 µl final reaction volume containing 5 µl of MyTaq Buffer 10X (Bioline), 1 µl of each primer [10 µM], 0.2 µl of MyTaq polymerase (Bioline) and 1.5 µl of DNA concentrated approximately 50 ng/µl. PCR reaction was performed as follow: a first denaturation step at 94 °C for 5 min followed by 35 cycles at 94 °C for 30 s, 60 °C for 30 s, 72 °C for 30 s and a final elongation step at 72 °C for 7 min. PCR amplified products were loaded in 1.2% agarose gels, visualized in the BioRAD transilluminator (BioRAD) and then digested by using a specific restriction enzyme. Five µl of the PCR products were digested using 0.5 µl of the enzyme *Bst*NI at 37 °C for 2 h, by adding 12 µl of distilled water and 2.5 µl of the specific buffer in a final volume of 20 µl. The resulting fragments were then separated on 3% agarose gels to distinguish between determinate and indeterminate genotypes.

### Marker Assisted Backcross (MABC) program

The backcross breeding program started in the summer of 2021: the determinate E42 parent was manually crossed with the five indeterminate donor parents in all possible combinations to obtain ten indeterminate F_1_ hybrids. The hybrids were used in the backcross schemes with the recurrent parent E42, carrying out two generations per year in 2022 and 2023 and thus reaching the BC_4_ progenies in August 2023. At each generation, the marker targeting the SP locus was used to select the heterozygous (*SPsp*) indeterminate plants and discard the homozygous determinate (*spsp*) ones. To fix the indeterminate plant habitus trait, the heterozygous BC_4_ progenies were self-pollinated and the BC_4_S_1_ homozygous for the dominant *SP* allele were selected in March 2024.

### Phenotypic evaluation of BC_4_S_1_ progenies

The BC_4_S_1_ combinations and their parental lines were evaluated for phenotypic traits during the 2024 summer season. The seeds were sown in seed trays in a greenhouse during the third week of March and transplanted to an open field (OF) in Acerra (Campania, 40°57’56.8” N, 14°25’33.9” E) at the end of April. The plants were grown in a completely randomized block design, with six replicates of each genotype and ten plants per replicate, and were managed in accordance with the standard cultivation practices adopted in the experimental area. This included manual pruning to remove lateral shoots and maintain a single main stem throughout the plant growth. At the beginning of June, a PVC plastic tunnel (PT) was used to cover three replicates per genotype to ensure higher temperature conditions. The plastic tunnel was made of a PVC structure covered with transparent polyethylene film. In addition, climatic data were recorded throughout the growing season using a GPS-6 temperature and humidity data logger (Elitech Innovation Preceding All, London, UK).

#### Evaluation of yield-related traits

Five phenotypic traits associated with yield performance were evaluated: the number of flowers per inflorescence (NFL), fruit set (FS), the total number of fruits per plant (TNF), fruit weight (FW) and yield per plant (YP). NFL was evaluated by counting the number of flowers per inflorescence on ten inflorescences per replicate, from the second to the fifth truss. FS was evaluated by counting flowers that set fruit out of all the flowers of each inflorescence on ten inflorescences per replicate, from the second to the fifth truss. FW was measured by weighing at least 30 fruits collected from all plants in the replicate, then dividing this value by the total number of fruits used. TNF was calculated by summing the number of fruits collected from all the plants in the three replicates. This value was then divided by the total number of plants. Finally, YP was assessed by taking the total weight of all the fruit collected and dividing it by the total number of plants in each replicate. The last two parameters were determined at the red ripe fruit stage.

#### Evaluation of fruit quality traits

Five fruit quality traits were evaluated: firmness, total soluble solids content (TSSC), titratable acidity (TA), pH and Fruit area. To perform a qualitative analysis, ten fruits at the red ripe stage were harvested from each replicate of each genotype, when full external ripening and uniform coloration was achieved, in accordance with the standard tomato maturity stage used for quality assessments. Firmness was measured using a penetrometer device with 0.6 mm nominal diameter of sensor (PCE-PTR 200, FL, USA). The TSSC, TA and pH were evaluated as reported by Olivieri et al. (2021). The Fruit area was assessed after scanning the fruits with the ImageJ analyzer (Schneider et al. 2012).

### Assessment of heat tolerance

To assess the physiological response of the BC₄S₁ lines to the contrasting thermal conditions (OF-*vs*-PT), the quantum yield of photosystem II (QY), hydrogen peroxide, lipid peroxidation and phenols content were evaluated. Chlorophyll fluorescence measurements were performed on healthy, fully expanded leaves of E42 and the BC₄S₁ lines grown under OF and PT conditions. Measurements were carried out on nine plants per genotype and environment, selecting leaves of comparable physiological age and position on the plant. The effective quantum yield of photosystem II (QY) was measured using a portable pulse-amplitude-modulated fluorometer, FluorPen FP 110 (Photon Systems Instruments, Drásov, Czech Republic). Measurements were performed on light-adapted leaves using the QY protocol provided by the manufacturer. Measurements were taken on the same leaf region, avoiding the main veins, and under the prevailing light conditions of each cultivation environment. At the end of the experimental period, leaf samples were collected from biological replicates of each genotype and growing condition, immediately frozen in liquid nitrogen and stored at − 80 °C. Frozen tissue was ground to a fine powder under liquid nitrogen to prevent metabolite degradation. Malondialdehyde (MDA) and total phenolic content (TPC) were quantified from fresh leaf tissue according to the Rainbow protocol described by López-Hidalgo et al. (2021). MDA was used as an indicator of membrane lipid peroxidation, whereas TPC was evaluated as a component of the non-enzymatic antioxidant response. Hydrogen peroxide (H_2_O_2_) content was determined according to Alexieva et al. (2001). Absorbance was measured at 390 nm, and H_₂_O_₂_ concentration was calculated using a standard curve prepared with known H₂O₂ concentrations. All biochemical parameters were expressed on a fresh-weight basis.

### Statistical analysis

Statistical analysis included:1) the Chi-square (χ²) test to evaluate the expected segregation ratio (1:1) obtained from each BC progeny; 2) the Student’s t-test to compare the performances in both environment (OF-vs.-PT); 3) one-way ANOVA and post-hoc Duncan tests to evaluate the parents and BC_4_S_1_ performances in each condition. All statistical analyses were performed using IBM SPSS v. 29 (IBM Corp., Armonk, NY, USA).

### Genomic analysis

Genomic analyses were conducted on five plants of each BC_4_S_1_ line and two plants of each parental line. DNA was extracted from leaf samples using the Qiagen DNeasy plant mini kit following the standard protocol. The DNA was then used for sequencing with the ddRAD protocol using Illumina sequencers (Novaseq6000). Libraries were prepared as reported in Olivieri et al. (2020), following DNA digestion with the enzymes *Mbo*I and *Sph*I. The raw FASTQ files were quality checked by using fastQC software (Andrews et al. 2010). Then, they were quality-filtered, clipped and trimmed using Trimmomatic (Bolger et al. 2014) v.0.39 (http://www.usadellab.org/cms/?page=trimmomatic) using the default parameters. Paired reads were aligned with the *Solanum lycopersicum* reference genome (Tomato Genome version SL4.0, available at the Solgenomics Network, www.solgenomics.net) using Bowtie-2 (Langmead and Salzberg 2012) with the default parameters. The resulting SAM and BAM files were sorted, de-duplicated and indexed with Samtools (Li et al. 2009). Variant calling was performed using BCFtools mpileup (Danecek et al. 2021) with the default parameters. The filtering step for variants detection was conducted using VCFtools (Danecek et al. 2011), setting the following parameters: minQ = 30 and minimum mean of depth of coverage (min–mean DP) = 15. The distribution of the SNPs across the chromosomes was estimated using the snpden function of VCFtools (1 Mb non-overlapping windows). Variants were annotated using SnpEff software (Cingolani et al. 2012), as reported by Graci et al. (2023).

## Results

### Marker Assisted Backcrossing

The backcross schemes were speeded up by selecting the heterozygous indeterminate plants of each progeny using the SP_CAPS marker built in the present work (Fig. S2A). The primers (primer forward: GGTAGAGTGATTGGTGAAGTTG; primer reverse: CATTTCCCCTTCCAAAGTAAAC) were designed to amplify a DNA fragment including the SNP mutation found by Pnueli et al. (1998) on the second exon of the SP gene and corresponding to a substitution of a timine (T) for a cytosine (C) at position 43,636,571 (Tomato Genome version SL4.0). The presence of T controls the determinate plant habitus, while the C is responsible for the indeterminate one. The primer forward was designed from positions 43,636,145 to 43,636,166, while the primer reverse mapped within positions 43,636,662 and 43,636,683. The PCR analysis performed using these two primers allowed to amplify a fragment of 539 bp in all the DNA samples extracted from both determinate and indeterminate plants. After digestion with *BstNI* (CCWGG) restriction enzyme, two fragments of 109 bp and 430 bp were produced from the indeterminate plants while the fragment of 539 bp was undigested from the determinate ones (Fig. S2B).

**Fig. 1:**
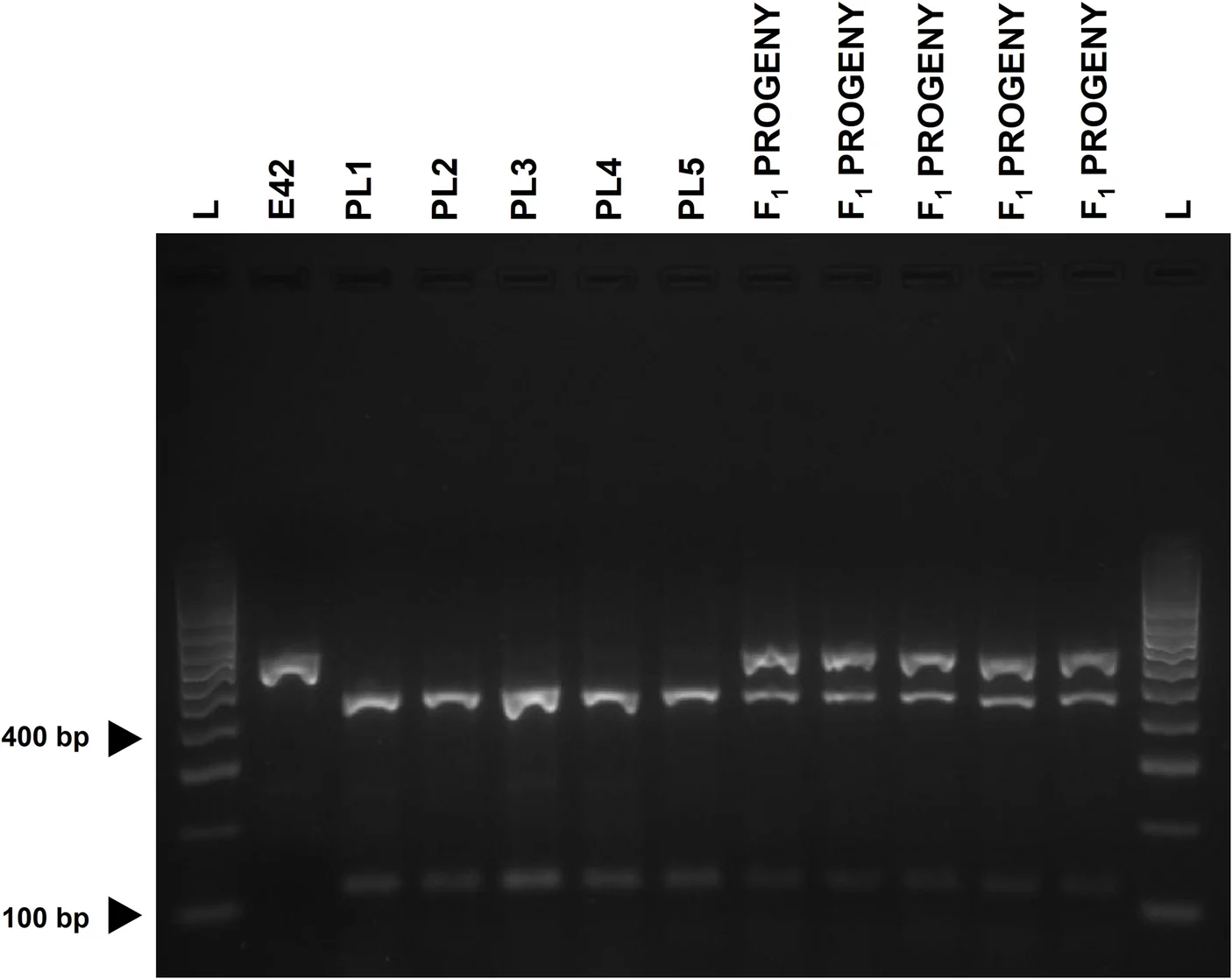
DNA tomato samples amplified using the SP_CAPS primers and digested by the *BstNI* restriction enzyme (3% agarose gel). The molecular marker allowed to distinguish homozygous indeterminate plants *SPSP* (PL1, PL2, PL3, PL4 and PL5), homozygous determinate plants *spsp* (E42) and heterozygous *SPsp* F_1_ progenies. L = 1 kb ladder

This marker was used to screen F1 hybrids and BC progenies during the backcross scheme (Fig. 1). The results of the molecular screening conducted during the backcross program are reported in Table S2. The chi square statistical test evidenced that the expected 1:1 ratio between the determinate and indeterminate plants was observed in all the backcross progenies, except than in the BC_2_ progeny of the E42-10 combination, where all the tested plants were found to be homozygous determinate (*spsp*) probably due to contamination of the seed batch. For this reason, the E42-10 backcross progeny was eliminated from the experiment.

### Phenotypic evaluation of BC_4_S_1_ progenies

**Fig. 2:**
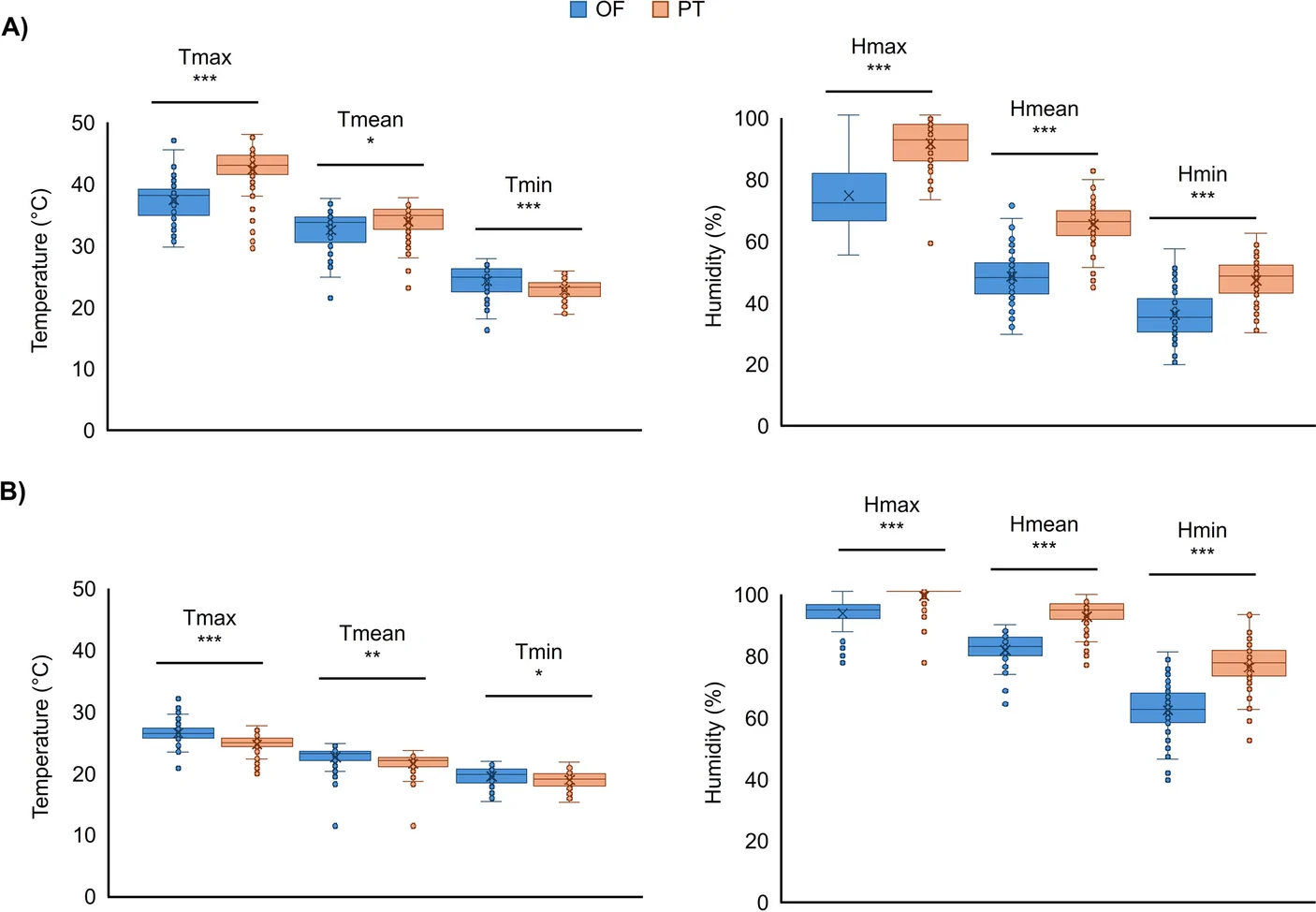
Temperature and relative humidity variations observed in open field (OF) and under plastic tunnel (PT) environments during the 2024 summer season in Acerra. The boxplot represents the comparison of maximum, mean and minimum temperatures (T) and relative humidity (H) recorded during the (A) day (from 7:00 a.m. to 8:00 p.m.) and (B) night (from 8:00 p.m. to 7:00 a.m.). The Student’s t-test was applied to evidence the significance of differences between the temperatures recorded in the two environments (shown above the graph. ns: not significant; *: *p* < 0.05; **: *p* < 0.01; ***: *p* < 0.001)

The performances of the nine BC_4_S_1_ progenies grown in OF and PT were assessed, since these indeterminate types could be grown in both the conditions (Fig. 2). Temperatures were recorded during the whole cultivation season in the two different environments. During the day, maximum temperatures always exceeded the critical threshold of 32 °C in both the environments (mean values of 37.3 °C in OF and 42.1 °C in PT). Also, the recorded mean day temperatures were high, around 32.5 °C in OF and 33.9 °C in PT. When considering the night temperatures, maximum and mean temperatures were higher than the critical threshold of 21 °C. Considering the relative humidity, the two environments displayed clear significant differences, with highest values in PT compared to OF.

**Fig. 3:**
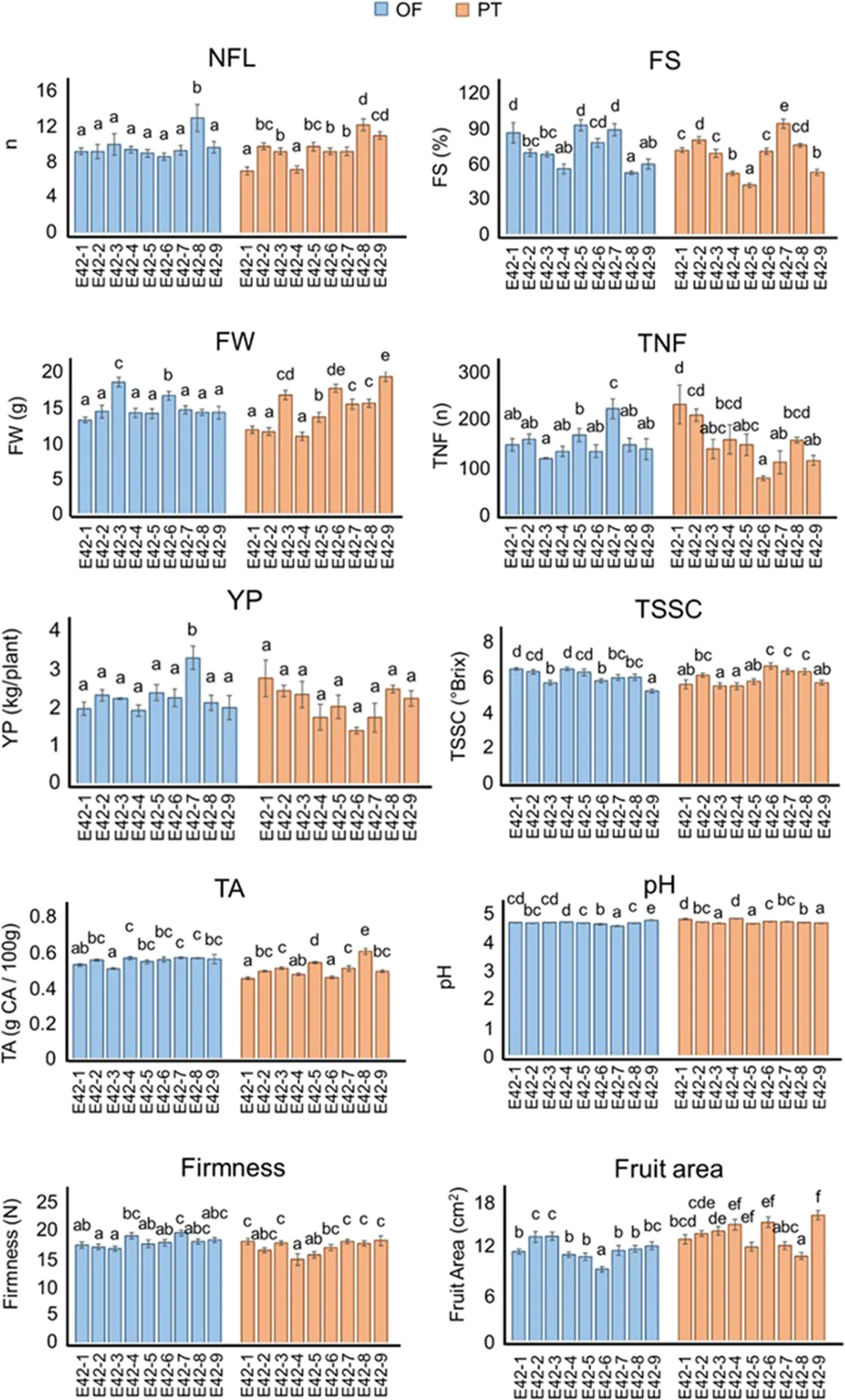
Yield-related and fruit quality trait performances of the nine BC_4_S_1_ progenies evaluated in open field (OF) and under plastic tunnel (PT) environments during the 2024 year. The traits evaluated are: Total number of flowers per inflorescence, NFL; Fruit set, FS; Fruit weight, FW; Total number of fruits per plant, TNF; yield per plant, YP; Total soluble solid content, TSSC; Titratable acidity, TA; pH; Firmness; Fruit area. Letters indicate significant differences among genotypes within each growing condition based on Duncan’s post-hoc test at *p* < 0.05. Bars represent mean values ± standard error

The performances of the nine BC_4_S_1_ progenies evaluated for 10 traits in the two environments are fully reported in Table S3 and as graphics in Fig. 3. Considering the yield-related traits, NFL, FW and YP were the most consistent traits observed among all the BC_4_S_1_ lines. Specifically, all the lines exhibited YP values comparable and usually higher than 2 kg/plant, except than E42-7 for which the recorded value in OF (3.3 kg/plant) was significantly higher in comparison with the other lines. By contrast, differences were recorded for the other traits, where FS ranged between 52.2% (E42-8) and 92.2% (E42-5) in OF and between 51.8% (E42-4) and 93.7% (E42.7) in PT; TNF ranged between 120.9 (E42-3) and 226.4 (E42-7) in OF and between 79.2 (E42-6) and 234.9 (E42-1) in PT. Focusing on the fruit quality traits, all the lines showed almost comparable TSSC (around 6 °Brix), TA (around 0.5), pH (around 4.5) and Firmness (higher than 15 N) with slight differences. The Fruit area was more variable, ranging from 9.1 cm^2^ (E42-6) to 13.3 cm^2^ (E42-3) in OF and from 10.7 cm^2^ (E42-8) to 15.9 cm^2^ (E42-9) in PT.

### Physiological and biochemical responses under high temperatures

**Fig. 4:**
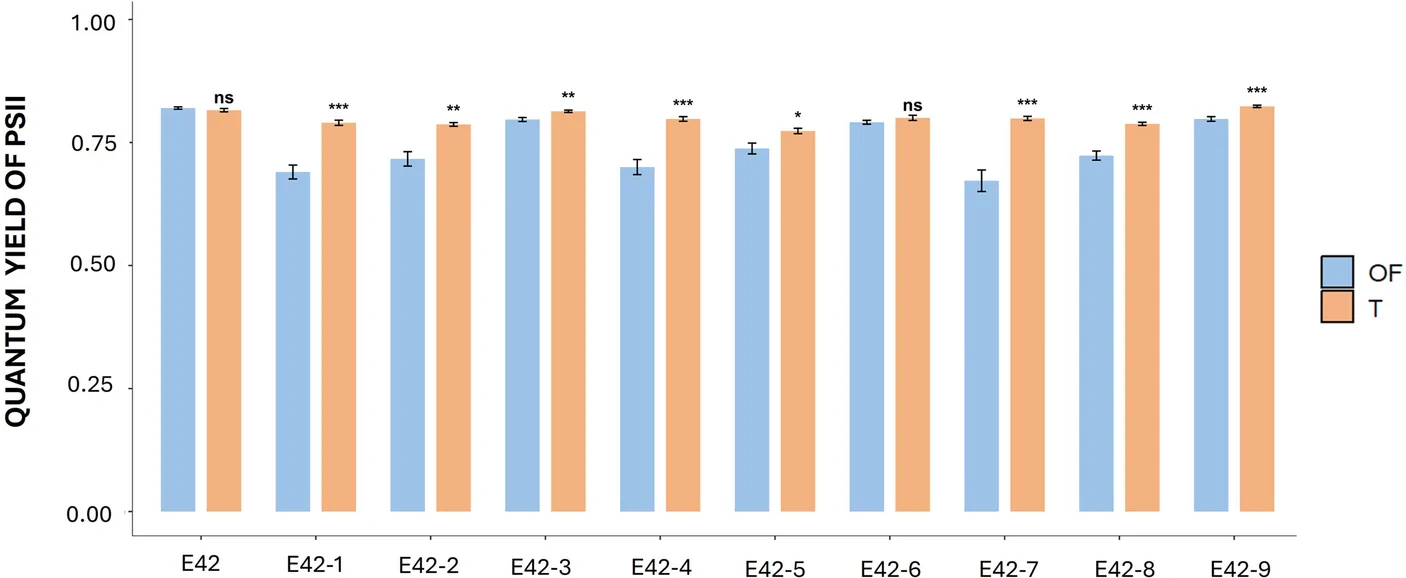
QY of the nine BC_4_S_1_ progenies and the parental E42 evaluated in open field (OF) and under plastic tunnel (PT) environments during the 2024 year. Differences between OF and PT conditions within each genotype were assessed using Student’s *t*-test. Data are presented as mean values ± standard error. Statistical significance was indicated as follows: ns, not significant; *, *p* < 0.05; **, *p* < 0.01; and ***, *p* < 0.001

To evaluate the physiological response to high temperatures of E42 and the BC₄S₁ progenies, the quantum yield (QY) was measured in OF and PT (Fig. 4). QY values recorded in OF were generally close to the expected range for leaves (0.7–0.8) with a well-functioning photosynthetic apparatus, suggesting that plants grown in the field did not exhibit severe PSII photoinhibition. Furthermore, QY remained stable or increased in most BC₄S₁ progenies under PT compared to OF conditions, with notable increases observed in nearly all genotypes except E42 and E42-6. Additional biochemical analyses revealed genotype-dependent responses in traits related to oxidative stress and antioxidants (see Table S4). The H_₂_O_₂_ and MDA content varied among the BC₄S₁ progenies. While in several cases the values under PT were comparable to or lower than those recorded under OF, in other cases a significant increase was observed. TPC also exhibited substantial genotype-dependent variation: some lines accumulated higher levels of phenolic compounds under PT, while others exhibited reduced or unchanged levels.

### Phenotypic recovery assessment of BC_4_S_1_ progenies

**Fig. 5:**
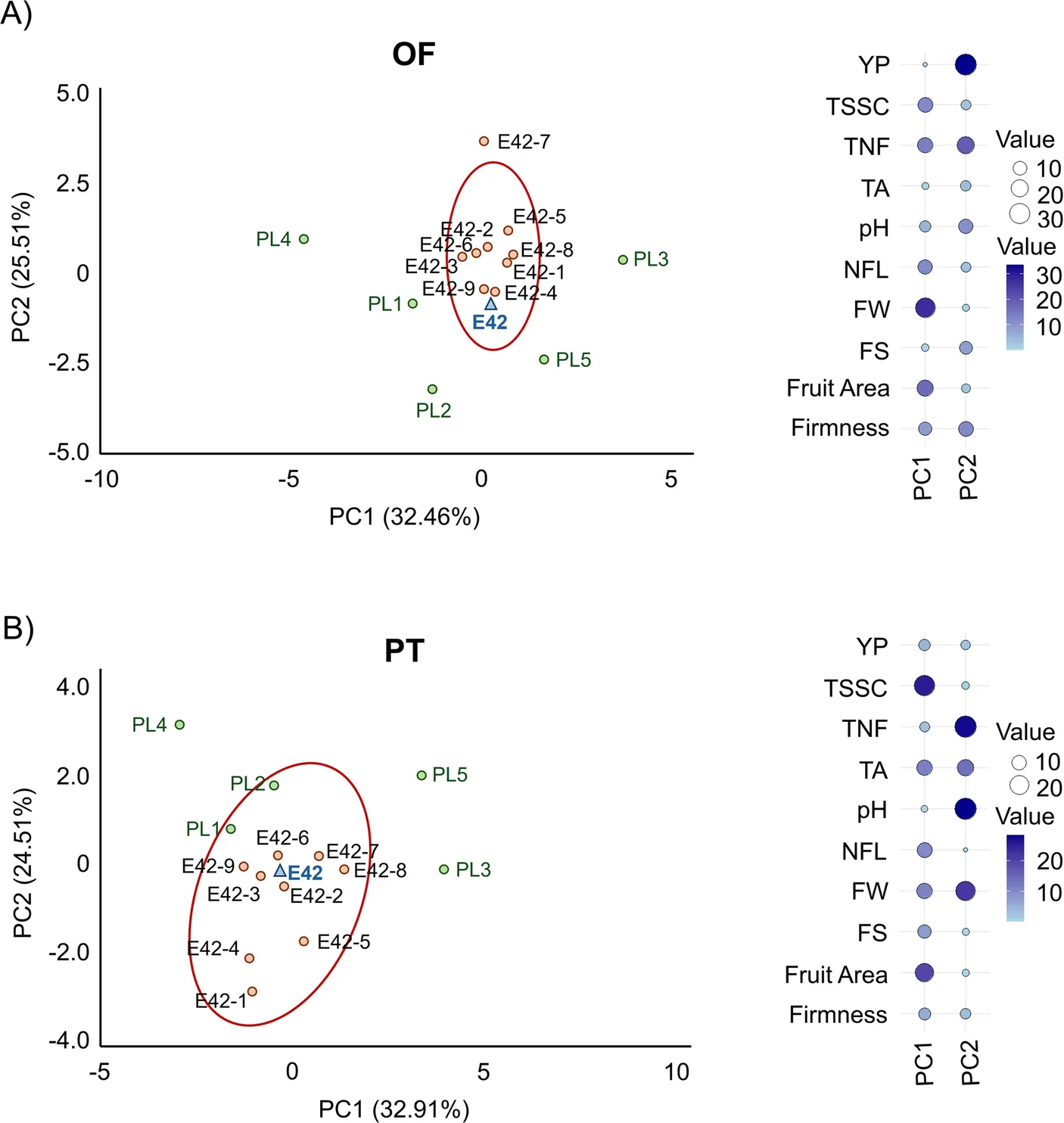
Principal Component Analysis (PCA) conducted on ten traits recorded from the nine BC_4_S_1_ progenies and their parental lines evaluated in (A) open field (OF) and (B) under plastic tunnel (PT). The analyzed ten traits are: number of flowers per inflorescence (NFL), fruit set (FS), fruit weight (FW), total number of fruits per plant (TNF), yield per plant (YP), firmness, total soluble solid content (TSSC), pH, titratable acidity (TA) and fruit area. Blue triangles indicate the recurrent parent E42, green spots the donor parents (PL1, PL2, PL3, PL4, PL5), orange spots the BC_4_S_1_ progenies (from E42-1 to E42-9). The clusters of lines resulting from PCA are grouped by red circles. The contribution of each trait for both PC1 and PC2 plotted are also reported, where higher contributions are represented by larger circles and darker blue colors

To assess the recovery of the E42 phenotype in each backcross progeny, the data of the nine BC_4_S_1_ lines in comparison with their parents (Table S5) were explored, and a principal component analysis (PCA) was conducted. Results showed that almost all the BC_4_S_1_ lines clustered with E42 in both the environments (Fig. 5), thus evidencing their high phenotypic similarity. In most cases, the donor lines fall outside the circle that groups BC_4_S_1_ progenies and the parent E42, thus evidencing clear differences for the evaluated traits between the two parents of each cross combination. Remarkably, E42-7 was the only line falling outside the cluster under OF environment.

### Genomic recovery-assessment of BC_4_S_1_ progenies

The VCF file generated by the SNP calling procedure included 532,412 high quality variants. Firstly, the divergence between E42 and the other parental lines was investigated, considering the total homozygous polymorphic variants between each pair of parents (Table 1).

**Table 1:** Genomic divergence of E42 from the other parental lines (PL) and from the BC_4_S_1_ progenies, estimated through ddRAD sequencing data

Code	Divergence (%)
PL1	21.42
PL2	26.24
PL3	34.11
PL4	26.78
PL5	25.00
E42-1	0.01–0.74
E42-2	0.01–0.02
E42-3	0.01–0.21
E42-4	0.01–0.02
E42-5	0.01–0.02
E42-6	0.01–0.02
E42-7	0.02–0.03
E42-8	0.01–0.02
E42-9	0.01–0.02

**Fig. 6:**
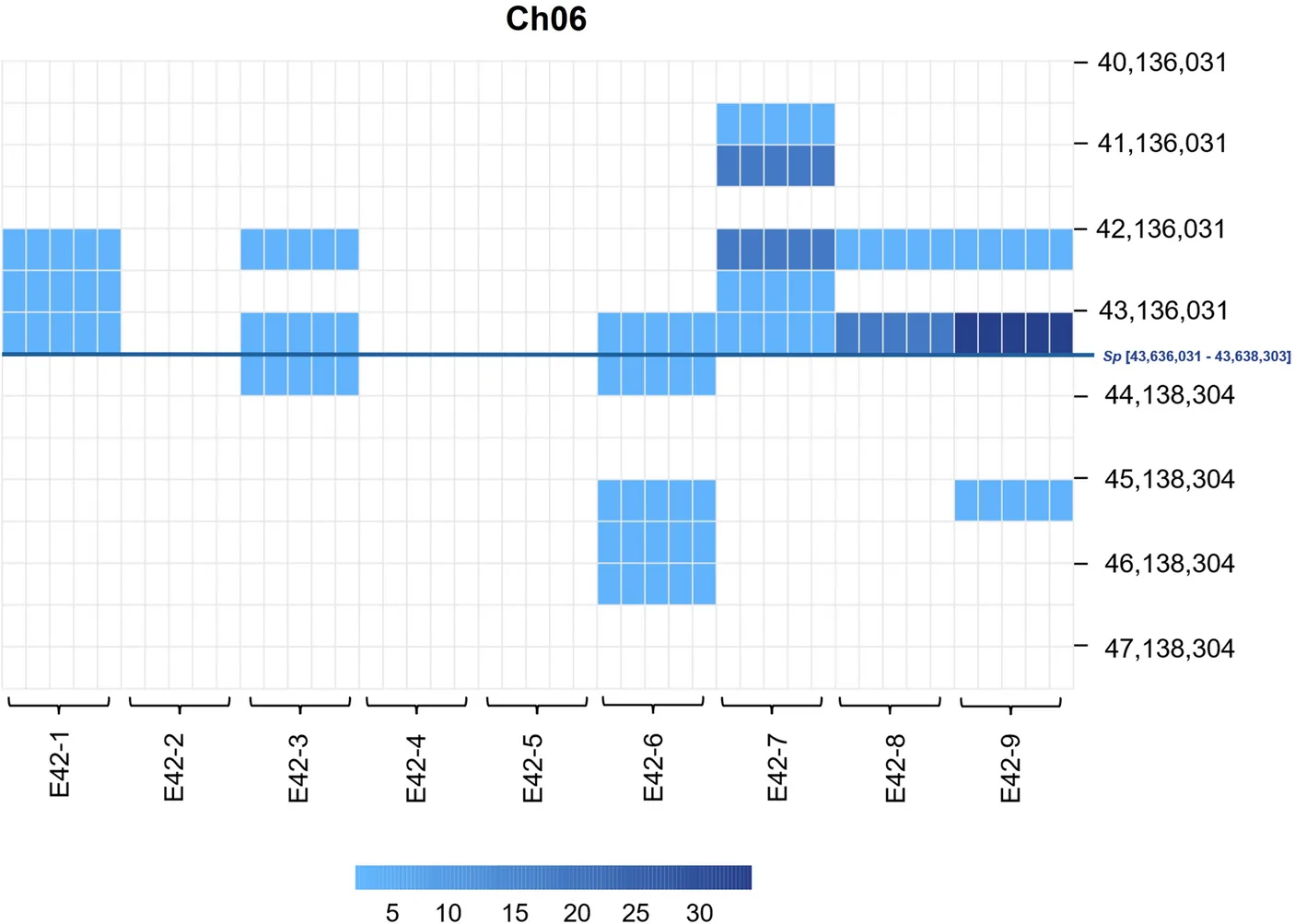
Heatmap showing the SNPs specifically deriving from the donor parents (blue) in the genomic region spanning the *self-pruning* gene (*Sp*) on chromosome 6. For each BC_4_S_1_ line, five plants were sequenced. Each column of the heatmap represents one plant. The analysis was conducted considering a windows size of 1 Mb, arbitrarily chosen. The blue line pointed by the arrow highlights the position where the *self-pruning* gene maps

Results evidenced that the divergence values between E42 and the other parents ranged between 21.4% (compared to PL1) and 34.1% (compared to PL3). The VCF file was then splitted in nine datasets, one for each BC_4_S_1_ progeny, to assess the percentage of the recurrent parent recovery based on the % of divergence observed between E42 and each PL line. Specifically, the BC_4_S_1_ lines were compared with both the parental lines (Table S6): in most cases (between 60.8% and 79.3%) there were not polymorphisms between the parents. When considering SNPs between the parents only a few of them (0.01% – 0.74%) were still derived from the donor parental line (Table 1), thus evidencing a high recovery of the recurrent genome. When looking at the distribution of variants specific of the donor parents (Fig. S3), results evidenced the presence of a few genomic regions mapping on chromosomes 1, 4, 6 and 7. Particularly, considering chromosome 6 in the region where the gene SP maps (between positions 43,636,031 and 43,638,303, Fig. 6), three BC_4_S_1_ lines (E42-3, E42-6, and E42-9) showed alleles of the donor around the genomic region of the SP gene. E42-1, E-42-7 and E42-9 showed polymorphisms from the donors only in the region up to the introgressed gene, with a variable number of genes involved, whereas the lines E42-2, E42-4 and E42-5 did not show polymorphisms from the donors, at least for the SNPs detected by the ddRAD sequencing technology used.

Details about polymorphic gene mapping in the region that spans the gene SP are reported in Fig. S4. E42 and PL3 parental lines presented several polymorphisms between positions 43,136,031 and 43,636,031, with 25 and 39 variants affecting 11 and 17 genes, respectively. Interestingly, E42-6 and E42-7 displayed opposite trends. E42-6 evidenced six variants exclusive of PL5 and affecting six genes, while the upstream region showed 35 genes with 85 variants exclusive of E42. E42-7 evidenced linkage drag upstream the SP gene with 54 variants exclusive of PL5 and affecting 25 genes, while the downstream region showed 12 genes with 22 variants exclusive of E42. When looking at the annotation of genes around the SP gene deriving from the donor parents (Table 2), nine genes mapping on chromosome 6 displayed polymorphisms with a MODERATE or HIGH impact on the protein sequence. Most of these polymorphisms (six variants in five genes) were observed in E42-7, whereas a lower number (three variants in three genes) were observed in E42-8 and E42-9. Finally, only one variant in one gene was evidenced in E42-1 and E42-6, respectively.

**Table 2:** List of genes showing variants with impact on the proteins derived from the donor parent around the introgressed SP gene. The gene ID, its position, the impact and effect of the variants, and the BC_4_S_1_ line showing the polymorphisms are reported, together with ITAG4.1 gene annotation (Solgenomics.net)

Gene	POS	Impact	Effect	BC_4_S_1_ lines	Annotation ITAG4.1
*Solyc06g070950*	41,232,022	MODERATE	conservative inframe insertion	E42-7	ABC transporter A family member 3
*Solyc06g071300*	41,543,346	MODERATE	disruptive inframe deletion	E42-7	AGAMOUS-like 98
*Solyc06g072650*	42,471,350	MODERATE	missense variant	E42-7	Small auxin up-regulated RNA61
*Solyc06g072830*	42,576,411	MODERATE	missense variant	E42-7	Tudor/PWWP/MBT superfamily protein
	42,576,521	MODERATE	missense variant	E42-7	
*Solyc06g073030*	42,665,484	HIGH	frameshift variant	E42-1, E42-7	Stomatin family protein
*Solyc06g073970*	43,386,979	MODERATE	missense variant	E42-8, E42-9	Core-2 i-branching beta-n-acetylglucosaminyltransferase family protein
*Solyc06g074270*	43,578,572	MODERATE	missense variant	E42-8, E42-9	The fantastic four family
*Solyc06g074280*	43,584,845	MODERATE	missense variant	E42-8, E42-9	Ankyrin repeat-containing protein
*Solyc06g082730*	46,021,083	MODERATE	missense variant	E42-6	Cytochrome P450

## Discussion

Plant breeders can help farmers to increase food production and quality by obtaining new plant material better adapted to current climate changes (Bradshaw 2017; Aliberti et al. 2020). Nowadays, the selection of thermotolerant tomato genotypes represents a tough challenge to face the threats of abiotic stresses in a general view of food security and environmental sustainability (Ayenan et al. 2021; Junior et al. 2022). Our recent research led to select the thermotolerant E42 genotype, since it showed a stable response to HS in terms of both yield and fruit quality traits (Olivieri et al. 2020, 2021). In the present work, we decided to develop a backcross breeding program aimed at enhancing this genotype for the fresh market by converting its plant habitus from determinate to indeterminate. Indeed, the fruit type and quality, its fruit truss and the jointed pedicel make E42 more eligible for the fresh market, and an indeterminate growth habit would ensure a scalar fruit maturation, more suitable for this type of market. The breeding work realized to reach the mentioned objective was assisted by two categories of molecular markers, one single locus CAPS and a high number of SNPs detected by the high throughput ddRAD genomic platform. The CAPS marker was designed to target the SNP found by Pnueli et al.(1998) on the SP gene, responsible for the habitus trait. Indeed, the use of molecular markers for the foreground selection (Osei et al. 2018) allowed identifying heterozygous genotypes *SPsp* to be backcrossed at each generation. This selection offers many advantages such as (I) the early stage of screening due to the possibility to conduct DNA marker analysis on any tissue; (II) the reduced number of plants to grown by maintaining only the selected ones, and (III) the absence of influence from the environment during the selection (Hasan et al. 2021). Since these advantages, molecular markers have been widely used in backcross programs to speed up the selection in many species. Kim et al.(2021) developed breeding lines of rice with low amylose content by applying MABC to backcross populations using KASP markers. In another work, Bharadwaj et al.(2021) enhanced drought tolerance of three elite Indian chickpea cultivars by using a MABC approach involving SSR markers and introgressing the “QTL-hotspot” region that harbors quantitative trait loci (QTLs) for several drought-tolerance related traits. Also in tomato, many cases are reported in which the MABC strategy has been successfully exploited to transfer single genes in elite varieties, mainly including resistance genes to pathogens (reviewed in Tiwari et al. 2022; Du et al. 2025).

Nine BC_4_S_1_ indeterminate progenies were obtained after selfing the selected BC_4_ genotypes. These were evaluated for both productive and fruit quality parameters under high temperatures in both OF and PT. During the growing season, maximum temperatures were often higher than 32 °C, which is reported to negatively affect plant development and reproductive processes in tomato (Ibrahim 2016; Bhattarai et al. 2021). Despite these conditions, the BC_4_S_1_ lines performed well, with mean YP values of 2.3 and 2.1 kg/plant in OF and PT, respectively. Moreover, they displayed good fruit quality, with an average TSSC value of 6 in both the OF and PT environments. To provide physiological support for the retention of heat-response characteristics in the BC₄S₁ lines, QY was evaluated under OF and PT conditions. Chlorophyll fluorescence-derived quantum yield is widely used to assess PSII functionality and provides a sensitive, non-destructive indicator of changes in photosynthetic performance under environmental stress (Maxwell and Johnson 2000; Baker 2008; Murchie and Lawson 2013). In tomato, fluorescence parameters have been successfully used to detect genotypic differences in photosynthetic stability and heat-stress response (Baker 2008; Bhattarai et al. 2021; Francesca et al. 2024). The maintenance or increase of QY observed in several BC₄S₁ lines under PT indicates that the high temperature did not cause a generalized impairment of PSII photochemistry. This interpretation is consistent with previous studies showing that maintenance of photosynthetic efficiency is an important component of heat resilience in tomato and other crops (Camejo et al. 2006), thus evidencing the thermotolerance of the BC_4_S_1_ lines. The biochemical measurements reported in Table S4 provided complementary information on oxidative pressure and antioxidant responses. H_₂_O_₂_ acts both as a reactive oxygen species and as a signaling molecule, whereas MDA is commonly used as an indicator of membrane lipid peroxidation. TPC contribute to non-enzymatic antioxidant defense and may increase in response to abiotic stress. In the present study, the responses of these parameters were strongly genotype dependent. Several BC₄S₁ lines maintained relatively low or unchanged H_₂_O_₂_ and MDA contents under PT, while increased phenolic accumulation in selected lines was consistent with activation of antioxidant-related protective mechanisms. Conversely, the marked accumulation of H_₂_O_₂_ or MDA in some genotypes indicates that the physiological response was not uniform across all BC_₄_ S_₁_ lines. Overall, these biochemical markers suggest that, while all genotypes perceived the imposed heat stress under PT, they activated different biochemical strategies involving ROS accumulation, membrane protection, and antioxidant responses. Therefore, the biochemical data were interpreted together with QY, fruit set, yield, and fruit-quality traits. The combined results show that most BC₄S₁ lines maintained favourable physiological and agronomic performance under the higher temperatures recorded in PT, supporting the retention of thermotolerance-associated characteristics of E42 after backcrossing.

To assess the recovery of E42 on each BC_4_S_1_ line, we investigated phenotype and genotype variability. The PCA analysis carried out on ten phenotypic traits evidenced a high similarity between almost all the BC_4_S_1_ lines and E42 (Bayles et al. 2005), except for E42-7, which exhibited higher YP when grown in OF. Recurrent genomic recovery was also estimated using Next Generation Sequencing (NGS) data obtained via ddRAD sequencing technology, to realize the background selection (Osei et al. 2018) at least at the final generation. The emergence of high-throughput sequencing technologies at the beginning of the 21st century allowed to identify millions of genome-wide SNPs useful for a high number of genetic studies (Kim et al. 2016, 2017; Phan and Sim 2017; Mancini et al. 2025). In the backcross scheme, 96.9% of recurrent genome is theoretically expected to be recovered in the BC_4_ progenies (Priyadarshan and Priyadarshan 2019). In accordance with this, most of the targeted genomic regions in our experiment were also found to have been recovered from the recurrent parent in all the BC_4_S_1_ lines. As expected, only a few percentages of variants (less than 1%) were kept from the donor, mainly around the SP gene, due to the phenomenon of linkage drag. Even though the percentage of genome explored by the ddRAD sequencing technology is generally very low (estimated between 0.1% and 5% in tomato), considering the SNPs detected between donor lines (PLs) and the parent E42, which cover all the 12 chromosomes of tomato, the recovery estimated on the initial divergence between parent pairs is consistent with the expected value for the entire genome. Indeed, the use of high-throughput genomic platforms, such as the GBS or RAD technologies, is well-documented in tomato MABC approaches and has enabled a high recovery rate of the recurrent genome in fewer generations than is achieved with traditional breeding approaches (Kim et al. 2019; Tripodi 2023). Since our initial crosses involved donor parents belonging to the cultivated *lycopersicum* background, which had been selected for heat tolerance and good fruit quality, we believed it would be possible to select some new genotypes as well - with certain characteristics even superior to those of the recurrent parent E42 - due to the interaction between the recurrent genome and specific regions of the donor. This was observed in genotype E42-7 for higher YP under OF conditions and in genotypes E42-3 and E42-6 for higher FW. For the same reason, we did not consider it useful to apply recombinant selection (Osei et al. 2018) to reduce linkage drag, since there are likely no genes with deleterious effects introduced by the donor, but there may be donor genes that exhibit excellent interactions with the recurrent genome, thus enhancing the phenotypic performances in the BC_4_F_1_ lines. It is precisely in this region that we have searched for donor alleles with moderate or high effect on the related proteins, which could explain certain specific phenotypes and represent improvements over E42.

Particularly, we assessed the donor alleles that were still present in a genomic region spanning across ± 3 Mb of the SP gene. It is well-known that, even following a high number of backcrosses, it is expected to find a remarkable fragment (till 10 cM) of the donor parent genome still linked to the gene target of the selection that may contain hundreds of genes (Tanksley et al. 1989; Cobb et al. 2019). Interestingly, the annotation of donor-derived variants within the linkage drag regions identified several genes whose predicted functions could potentially contribute to the phenotypic variability observed among the BC₄S₁ lines for some evaluated traits. We found a group of genes mapping 500 kb upstream the target gene showing variants introgressed from the donor PL3 parent in both the E42-8 and E42-9 lines. Among these, three genes (*Solyc06g073970*, *Solyc06g074270* and *Solyc06g074280*) showed missense variants that are predicted to affect the translated protein. Specifically, we focused our attention on the *Solyc06g074270* gene coding for the fantastic four family (FAF). This gene family is reported to be implicated in the regulation of shoot meristem size by modulating the CLAVATA 3 (CLV3)-WUSCHEL (WUS) feedback loop (Wahl et al. 2010). Specifically, previous studies have shown that FAF genes in tomato influence the transition of the shoot apical meristem from vegetative to reproductive growth by modulating the expression of florigenic genes such as Single Flower Truss (SFT) (Zhang et al. 2024). Moreover, there has been evidence to show that the FAF gene family responds to photoperiodic changes (Wahl et al. 2010; Torti et al. 2012; You et al. 2019). The variants affecting this gene may be responsible of the E42-8 and E42-9 phenotype, which is peculiar when compared to the other BC_4_S_1_ lines since they presented a flower morphology in accordance with the PL3 phenotype, resulting in a smaller flower, with sepals pointing backwards and a thinner anther cone (Graci et al. 2025). The E42-6 linkage-drag region contained a missense variant in *Solyc06g082730*, encoding a cytochrome P450. Plant cytochrome P450 enzymes participate in hormone metabolism, secondary-metabolite biosynthesis, fruit development and stress responses (Tang et al. 2024). Therefore, this gene is a promising candidate for variations in fruit or stress-related traits, potentially influencing the increased FW observed in the E42-6 line. Other authors have indeed reported the presence of QTLs for FW (fw6.2, fw6.3) in this chromosome (Ning et al. 2023; Feng et al. 2026), very close to the gene SP. In E42-7, a conservative in-frame insertion was identified in *Solyc06g070950*, encoding an ABC transporter. Plant ABC transporters mediate the movement of hormones, secondary metabolites and stress-related compounds, and some ABCB members participate in polar auxin transport (Wu et al. 2007). Therefore, this variant could potentially affect hormone distribution or stress responses in E42-7, although its specific role in yield, fruit set or plant architecture remains unknown. The same line carried a disruptive in-frame deletion in *Solyc06g071300*, annotated as *AGAMOUS-like 98*. AGAMOUS-like MADS-box transcription factors regulate flowering, floral meristem determinacy and floral organ identity (Schilling et al. 2018). This variant could therefore influence reproductive development in E42-7. In the same line a missense variant was also detected in *Solyc06g072650*, encoding *Small auxin up-regulated RNA 61*. SAUR genes are involved in auxin-dependent cell expansion and organ growth. Thus, this variant could affect floral or early fruit development, although functional redundancy within the SAUR family limits predictions based on annotation alone (Wu et al. 2012). Overall, *Solyc06g070950*,* Solyc06g071300*,* Solyc06g072650*,* Solyc06g074270* and *Solyc06g082730* represent the most plausible candidates among the genes carrying donor-derived impactful variants. Their predicted functions suggest possible roles in auxin transport and signaling, floral development, cell expansion, protein interactions, secondary metabolism and stress responses. Besides the genes located around the introgressed SP locus, two genomic regions retaining donor-derived variants were also detected in E42-7 on chromosomes 1 and 4. These regions map between 76 and 77 Mb on chromosome 1 and between 60 and 61 Mb on chromosome 4, and co-localize with QTLs related to yield (SL4.0ch01_68237408 and SL4.0ch04_62857466), number of fruits (FRN1.1_T2_16) and fruit set (FRS4.2_T3_16), as reported by Elazazi et al. (2024). From the PCA conducted on the yield-related traits in OF, we also observed that the E42-7 line falls outside the group including the BC_4_S_1_ and E42 genotypes due to its superior performances in terms of YP, TNF and FS. The highlighted polymorphic genomic regions may play a role in the E42-7 superior performances in comparison with the other lines that will be further investigated in the future.

## Conclusions

In conclusion, this study enabled us to adapt for the fresh tomato market the E42 genotype, which was previously selected for thermotolerance and high fruit quality. Overall, our work focused on converting the thermotolerant, determinate genotype E42 into an indeterminate type. We integrated the foreground selection at the SP locus in a MABC approach with detailed phenotypic assessment and ddRAD-based genomic analysis of advanced BC₄S₁ lines grown in OF and under PT. The resulting lines largely recover the E42 phenotype, maintain high levels of the recurrent genome and demonstrate stable performance with regarding yield and fruit quality traits under high temperature conditions. However, these results should be considered preliminary and require validation through additional multi-environmental and multi-year trials, to confirm the agronomic performance and robustness of the selected BC_4_S_1_ lines under different growing conditions, among which the higher-performing line E42-7 was identified. This indicates that converting the plant habitus did not compromise the desirable features that originally motivated the selection of E42. These BC₄S₁ lines therefore represent valuable pre-breeding material for fresh-market cherry tomatoes or small-fruited tomatoes adapted to Mediterranean-like environments. They can be used directly as parents in F₁ hybrid development and as donors of combined thermotolerance and fruit quality. It is also important to emphasize that the residual portions of the donor genome, evidenced through the ddRAD analysis, whether due to linkage drag or present on other chromosomes, introduced additional favorable traits into the BC_4_S_1_ lines beyond those of E42. In fact, the donors are all lines with a cultivated genome, previously selected for tolerance to high temperatures and good fruit quality. The investigation of these donor regions associated with good performance in the BC_4_S_1_ lines led to identify candidate genes with alleles that are favorable for specific traits. In the future, it will be important to functionally characterize the candidate genes identified in this study to better clarify their potential role in the observed phenotypic variation.

## Supplementary Information

Below is the link to the electronic supplementary material.

Supplementary Material 1

Supplementary Material 2

Supplementary Material 3

Supplementary Material 4

Supplementary Material 5

Supplementary Material 6

Supplementary Material 7

Supplementary Material 8

Supplementary Material 9

Supplementary Material 10

## Data Availability

The data presented in this study are available on request from the corresponding author.
