# Supplementary material for: Integrating marker-assisted backcross breeding and genomic analysis to obtain thermotolerant tomato genotypes for the fresh market
Source: Mol Breed. 2026 Oct 29;46(11):111. doi: 10.1007/s11032-026-01730-0 (PMC13719954; doi:10.1007/s11032-026-01730-0)
Supplement: Supplementary file 1 [file 11032_2026_1730_MOESM10_ESM.docx]

**Figure S1.** Fruit morphology of the recurrent and donor parental genotypes used in the backcross breeding program.
