# Supplementary material for: Integrating marker-assisted backcross breeding and genomic analysis to obtain thermotolerant tomato genotypes for the fresh market
Source: Mol Breed. 2026 Oct 29;46(11):111. doi: 10.1007/s11032-026-01730-0 (PMC13719954; doi:10.1007/s11032-026-01730-0)
Supplement: Supplementary file 2 [file 11032_2026_1730_MOESM2_ESM.docx]

| **G** | **E** | **MDA**  **(ng g^-1^ FW)** | **H_2_0_2_**  **(mmol g ^-1^)** | **Total Phenols**  **(µg mg^-1^ FW)** |
| --- | --- | --- | --- | --- |
| E42 | OF | 2437.85 ± 71.32 | 1.206 ± 0.027 | 0.596 ± 0.025 |
|  | PT | 1488.24 ± 79.32*** | 1.504 ± 0.040*** | 0.850 ± 0.054*** |
| E42_1 | OF | 1820.05 ± 90.96 | 2.365 ± 0.186 | 0.993 ± 0.009 |
|  | PT | 2349.39 ± 117.29*** | 1.165 ± 0.165*** | 0.563 ± 0.013*** |
| E42_2 | OF | 687.58 ± 43.48 | 1.927 ± 0.054 | 0.568 ± 0.108 |
|  | PT | 1823.29 ± 77.68*** | 1.432 ± 0.085*** | 1.180 ± 0.038*** |
| E42_3 | OF | 2147.37 ± 494.61 | 1.295 ± 0.073 | 0.469 ± 0.014 |
|  | PT | 1396.83 ± 94.08** | 1.644 ± 0.067*** | 1.201 ± 0.018*** |
| E42_4 | OF | 1484.21 ± 130.08 | 2.061 ± 0.037 | 0.857 ± 0.059 |
|  | PT | 2164.70 ± 320.42*** | 1.061 ± 0.066*** | 0.748 ± 0.027** |
| E42_5 | OF | 913.48 ± 102.23 | 1.221 ± 0.028 | 1.225 ± 0.025 |
|  | PT | 2252.93 ± 52.68*** | 3.745 ± 0.106*** | 0.486 ± 0.020*** |
| E42_6 | OF | 2263.17 ± 33.85 | 1.719 ± 0.047 | 0.475 ± 0.022 |
|  | PT | 2038.89 ± 100.54*** | 1.856 ± 0.080*** | 0.707 ± 0.103*** |
| E42_7 | OF | 1503.82 ± 52.90 | 1.609 ± 0.049 | 0.833 ± 0.024 |
|  | PT | 1850.73 ± 88.57*** | 1.699 ± 0.042*** | 0.611 ± 0.010*** |
| E42_8 | OF | 2488.43 ± 88.43 | 3.488 ± 0.067 | 1.230 ± 0.100 |
|  | PT | 1964.28 ± 75.47*** | 2.197 ± 0.035*** | 0.738 ± 0.030*** |
| E42_9 | OF | 3395.20 ± 72.28 | 2.486 ± 0.060 | 0.826 ± 0.034 |
|  | PT | 3176.98 ± 121.07** | 1.331 ± 0.033*** | 0.823 ± 0.111^ns^ |

**Table S4**. Biochemical responses of E42 and BC₄S₁ progenies under open-field (OF) and plastic-tunnel (PT) conditions. Values are reported as mean ± standard deviation. For each genotype, differences between OF and PT conditions were assessed using a Student’s *t*-test (ns, not significant; *, *p* < 0.05; **, *p* < 0.01; and ***, *p* < 0.001).
