# Supplementary material for: Integrating marker-assisted backcross breeding and genomic analysis to obtain thermotolerant tomato genotypes for the fresh market
Source: Mol Breed. 2026 Oct 29;46(11):111. doi: 10.1007/s11032-026-01730-0 (PMC13719954; doi:10.1007/s11032-026-01730-0)
Supplement: Supplementary file 3 [file 11032_2026_1730_MOESM7_ESM.docx]

.

**Figure S2** – Design and validation of the CAPS-SP marker. A) Schematic representation of the self-pruning (SP) gene structure. Blue and green boxes represent exons and introns, respectively, and orange boxes represent UTRs regions (Figure modified from Solgenomics, www.solgenomics.net). The SNP is located on the starting position of the second intron of the SP gene. The reported alignment between the determinate and indeterminate tomato genotypes evidence that the mutation is a change from thymine to cytosine. B) DNA tomato samples amplified using the SP_CAPS primers and digested by the *BstNI* restriction enzyme (3% agarose gel). The molecular marker allowed to distinguish the homozygous indeterminate plants *SPSP* (430 bp and 109 bp fragments) from the homozygous determinate ones *spsp* (539 bp fragment), L=1 kb ladder
