# Supplementary material for: Integrating marker-assisted backcross breeding and genomic analysis to obtain thermotolerant tomato genotypes for the fresh market
Source: Mol Breed. 2026 Oct 29;46(11):111. doi: 10.1007/s11032-026-01730-0 (PMC13719954; doi:10.1007/s11032-026-01730-0)
Supplement: Supplementary file 4 [file 11032_2026_1730_MOESM8_ESM.docx]

**Figure S3** - Heatmap representing the number of alleles derived from the donor parent (blue squares) on the 12 chromosomes of each BC_4_S_1_ line. The analysis was conducted considering a windows size of 1Mb.

**Figure S3 (continue)** - Heatmap representing the number of alleles derived from the donor parent (blue squares) on the 12 chromosomes of each BC_4_S_1_ line. The analysis was conducted considering a windows size of 1Mb.

**Figure S3 (continue)** - Heatmap representing the number of alleles derived from the donor parent (blue squares) on the 12 chromosomes of each BC_4_S_1_ line. The analysis was conducted considering a windows size of 1Mb.

**Figure S3 (continue)** - Heatmap representing the number of alleles derived from the donor parent (blue squares) on the 12 chromosomes of each BC_4_S_1_ line. The analysis was conducted considering a windows size of 1Mb.

**Figure S3 (continue)** - Heatmap representing the number of alleles derived from the donor parent (blue squares) on the 12 chromosomes of each BC_4_S_1_ line. The analysis was conducted considering a windows size of 1Mb.

**Figure S3 (continue)** - Heatmap representing the number of alleles derived from the donor parent (blue squares) on the 12 chromosomes of each BC_4_S_1_ line. The analysis was conducted considering a windows size of 1Mb.

**Figure S3 (continue)** - Heatmap representing the number of alleles derived from the donor parent (blue squares) on the 12 chromosomes of each BC_4_S_1_ line. The analysis was conducted considering a windows size of 1Mb.

**Figure S3 (continue)** - Heatmap representing the number of alleles derived from the donor parent (blue squares) on the 12 chromosomes of each BC_4_S_1_ line. The analysis was conducted considering a windows size of 1Mb.

**Figure S3 (continue)** - Heatmap representing the number of alleles derived from the donor parent (blue squares) on the 12 chromosomes of each BC_4_S_1_ line. The analysis was conducted considering a windows size of 1Mb.

**Figure S3 (continue)** - Heatmap representing the number of alleles derived from the donor parent (blue squares) on the 12 chromosomes of each BC_4_S_1_ line. The analysis was conducted considering a windows size of 1Mb.

**Figure S3 (continue)** - Heatmap representing the number of alleles derived from the donor parent (blue squares) on the 12 chromosomes of each BC_4_S_1_ line. The analysis was conducted considering a windows size of 1Mb.

**Figure S3 (continue)** - Heatmap representing the number of alleles derived from the donor parent (blue squares) on the 12 chromosomes of each BC_4_S_1_ line. The analysis was conducted considering a windows size of 1Mb.
