# Supplementary material for: Integrating marker-assisted backcross breeding and genomic analysis to obtain thermotolerant tomato genotypes for the fresh market
Source: Mol Breed. 2026 Oct 29;46(11):111. doi: 10.1007/s11032-026-01730-0 (PMC13719954; doi:10.1007/s11032-026-01730-0)
Supplement: Supplementary file 5 [file 11032_2026_1730_MOESM9_ESM.docx]

**Figure S4** - Graphic representation of genes on chromosome 6 showing variants in the parental and in the BC_4_S_1_ lines. In black alleles common to the parental lines, in blue alleles with variants from E42, in red alleles with variants from the donor line. The position of genes in 14 genomic windows of 500,000 bases upstream and downstream the SP gene is shown, being this interval arbitrary chosen.

**Figure S4 (continue)** - Graphic representation of genes on chromosome 6 showing variants in the parental and in the BC_4_S_1_ lines. In black alleles common to the parental lines, in blue alleles with variants from E42, in red alleles with variants from the donor line. The position of genes in 14 genomic windows of 500,000 bases upstream and downstream the SP gene is shown.

**Figure S4 (continue)** - Graphic representation of genes on chromosome 6 showing variants in the parental and in the BC_4_S_1_ lines. In black alleles common to the parental lines, in blue alleles with variants from E42, in red alleles with variants from the donor line. The position of genes in 14 genomic windows of 500,000 bases upstream and downstream the SP gene is shown, being this interval arbitrary chosen.

**Figure S4 (continue)** - Graphic representation of genes on chromosome 6 showing variants in the parental and in the BC_4_S_1_ lines. In black alleles common to the parental lines, in blue alleles with variants from E42, in red alleles with variants from the donor line. The position of genes in 14 genomic windows of 500,000 bases upstream and downstream the SP gene is shown, being this interval arbitrary chosen.

**Figure S4 (continue)** - Graphic representation of genes on chromosome 6 showing variants in the parental and in the BC_4_S_1_ lines. In black alleles common to the parental lines, in blue alleles with variants from E42, in red alleles with variants from the donor line. The position of genes in 14 genomic windows of 500,000 bases upstream and downstream the SP gene is shown, being this interval arbitrary chosen.

.
